# The national registration and accreditation scheme

**DOI:** 10.1186/1757-1146-4-S1-I12

**Published:** 2011-05-20

**Authors:** Jason Warnock, Ebenezer Banful, Laurie Foley, Mark Gilheany, Anne-Marie Hunter, Cathy Loughry, Helen Matthews, Joan Russell, Paul Tinley

**Affiliations:** 1 Chair, Podiatry Board of Australia; 2 Board Members, Podiatry Board of Australia

## 

Australia is the first country in the world to have a national registration and accreditation scheme regulating health practitioners. All states and territories are part of the scheme, with Western Australia joining on 18^th^ October 2010; the other states and territories commencing on 1^st^ July 2010.

The theme of this conference is appropriately “Setting the Pace”. The Podiatry Board of Australia (PodBA) was worked feverishly since its first meeting on 20^th^ September 2009 to prepare for commencement day. The PodBA and the other nine national boards were challenged to prepare for the implementation of the “National Law” when the legislation had not been presented and passed though state and territory parliaments, when there were no offices and no staff for the support agency AHPRA (Australian Health Practitioners Regulation Agency) and when the Boards had no finances of their own.

The task has been enormous. Goodwill and faith in the process has been required of the community, governments, professions, accreditation organisations, educators, the state and territory boards and you the registrants. Thank you for your patience.

This presentation provides me with the opportunity to explain some the functions of the PodBA and how they will impact you. Topics will include:

• The Registration and Accreditation Scheme

• Registration

• Registration Standards

• Codes and Guidelines

• The Public Register

• Notifications

The PodBA has a small booth in the Exhibition Hall. Please come to the display and introduce yourself to the Jenny Collis (Executive Officer for PodBA) and other members of the PodBA.

